# Thrombocytopenia Secondary to Herpes Simplex Virus-2 Infection Successfully Treated by Acyclovir

**DOI:** 10.7759/cureus.12338

**Published:** 2020-12-28

**Authors:** Raheel S Siddiqui, Sofia Lakhdar, Chandan Buttar, Merjona Saliaj

**Affiliations:** 1 Internal Medicine, Icahn School of Medicine at Mount Sinai, New York City Health and Hospitals/Queens, Jamaica, USA

**Keywords:** thrombocytopenia, hsv-2, genital herpes, acyclovir

## Abstract

Thrombocytopenia is a common clinical condition associated with a wide variety of clinical conditions including infections, malignancy, medications, liver disorder, and autoimmune conditions, etc. The association between thrombocytopenia and herpes simplex virus (HSV) is reported only once in a case report dating back to 1978. We report a case of a 66-year-old female with generalized weakness, mechanical fall, genital ulcerations, and breast fold and genital area skin redness, warmth, and mild tenderness. Initial labs showed mild leukocytosis, normal platelet count, mild lactic acidosis, and urine analysis suggestive of urinary tract infection. The patient was started on broad-spectrum antibiotics. During the course of hospitalization, the patient developed severe thrombocytopenia with platelet counts dropping less than 40000/μL (normal range: 150,000-450,000/μL), and genital pain and ulceration worsened. The genital swab was sent which came back positive for the HSV-2 virus. Soon after the start of acyclovir for HSV-2 infection, the genital pain and ulceration improved and platelet counts gradually increased to 157,000/μL. Other causes of thrombocytopenia such as sepsis, heparin-induced thrombocytopenia, consumptive coagulopathy, medication-induced thrombocytopenia, immune thrombocytopenia, and thrombotic thrombocytopenic purpura were ruled out.

## Introduction

Thrombocytopenia is a common clinical finding associated with a wide variety of clinical conditions including infections, malignancy, liver disease, medications, pregnancy, autoimmune diseases, and disseminated intravascular coagulation, etc [1]. Most of the cases of thrombocytopenia are asymptomatic and rarely associated with severe bleeding. Platelets count below 30,000/μL is associated with mucocutaneous bleeding such as petechiae, skin purpura (dry purpura), and mucous membrane purpura (wet purpura) whereas platelets count less than 10,000/μL is associated with spontaneous intracranial bleeding and the patient should be considered for platelets transfusion [2,3]. The major causes of a low platelet count are divided into four major categories: pseudo thrombocytopenia due to clumping of the platelets in vitro, under production, sequestration, and destruction [4]. 

Herpes simplex virus-2 (HSV-2) infection is not a well-known cause of thrombocytopenia. The association between thrombocytopenia and HSV-2 infection was first reported in a case report in 1978 [5]. Since then literature is scarce about thrombocytopenia in HSV-2. We report a case of severe thrombocytopenia in a patient with active HSV-2 infection which improved after treatment with acyclovir.

## Case presentation

**Figure 1:**
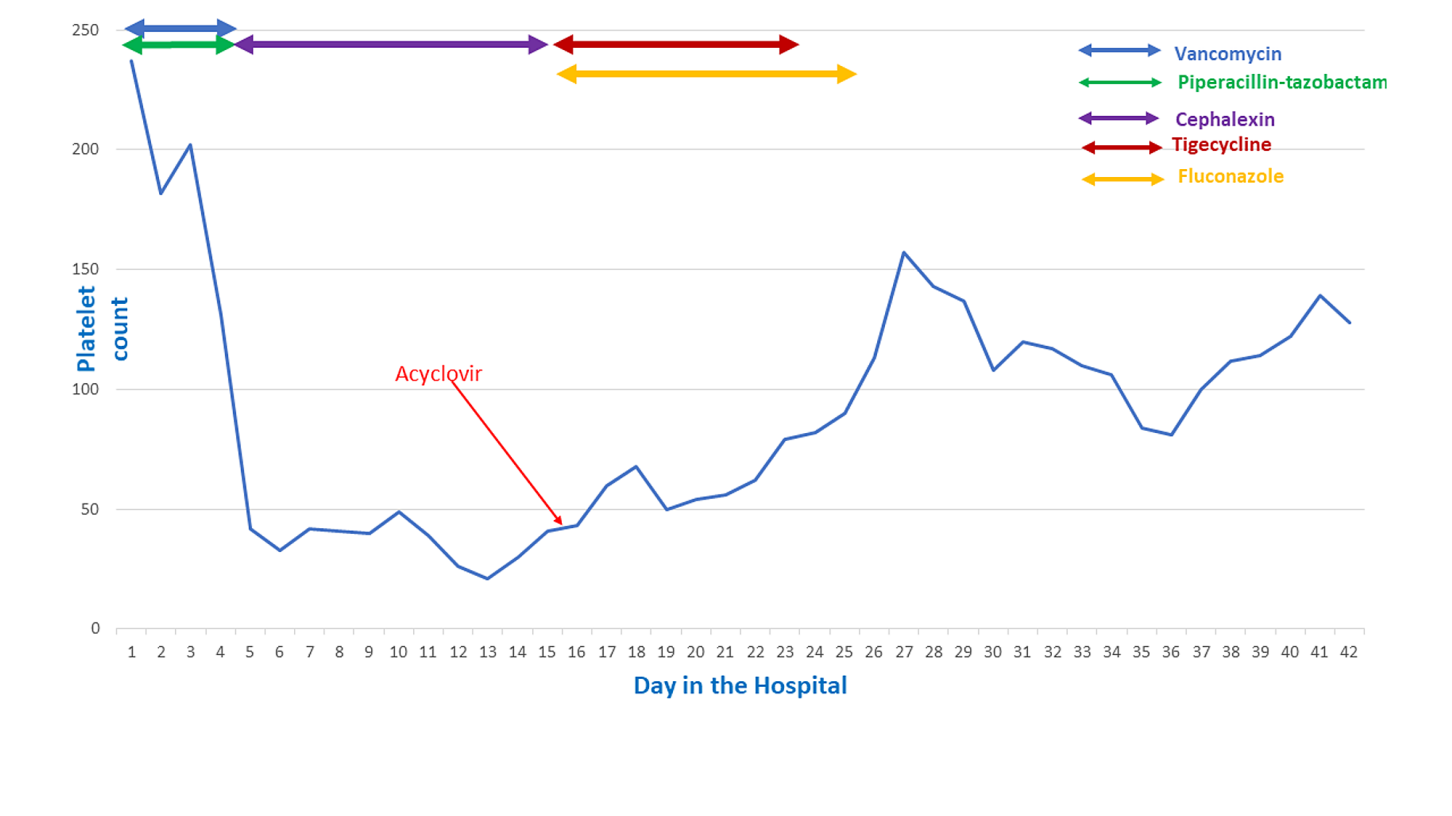
Trend of platelet count during the course of hospitalization.

A 66-year-old woman presented to the emergency room after a mechanical fall and complained of generalized weakness. The patient had pain all over the body, especially worse in the genital area. The patient had a history of multiple sclerosis for 10 years and was on a weekly interferon beta-1a injection. Vitals showed blood pressure of 88/62 mmHg, heart rate 96 beats per minute and temperature of 97.3 F. Physical examination showed erythema, warmth and tenderness of the skin under the breast folds. The skin around the sacral and genital area showed extensive redness and warmth and multiple ulcerative lesions. Baseline laboratory values on admission showed white blood cell (WBC) 14,030/μL (normal range: 4,800-11,000/μL) hemoglobin (Hgb) 11.9 g/dL (normal range: 12-16 g/dL), platelets 237,000/μL (normal range: 150,000-450,000/μL), blood urea nitrogen 54 mg/dL (normal range: 7-23 mg/dL), creatinine 1.52 mg/dL (normal range: 0.5-1.3 mg/dL), lactic acid 6.3 mmol/L (normal range: 0.5-2.2 mmol/L) and urine analysis suggestive of infection. Urine culture was contaminated and repeated urine culture after antibiotic therapy did not grow any microorganism. Blood cultures did not grow any organism. The patient was started on vancomycin and piperacillin-tazobactam along with intravenous hydration under the impression of sepsis secondary to cellulitis and urinary tract infection. Subcutaneous heparin was given for deep venous thrombosis prophylaxis. The patient reported mild improvement in generalized weakness. On day 5 of the hospital admission, platelet count gradually dropped to 33,000/μL and WBC trended up to 19,240/μL. Fibrinogen level of 315 mg/dL (normal range: 200-393 mg/dL), international normalized ratio (INR) of 1.2, activated partial thromboplastin time (APTT) of 25.7 (normal range: 25.1-36.5 seconds) were noted. Peripheral smear did not show any schistocyte. Heparin, vancomycin, and piperacillin-tazobactam were stopped and the patient was started on cephalexin. Serotonin release assay and heparin-induced platelet antibodies were negative. Intravenous methylprednisolone and seven days of plasmapheresis failed to improve platelet count. Bone marrow biopsy showed 50%-60% cellularity with trilineage hematopoiesis. Fluorescent in situ hybridization (FISH) and flow cytometry on bone marrow aspirate were unremarkable and ADAMTS13 activity was within the normal range. The patient reported worsening of genital area pain and worsening of genital ulceration was noted on physical examination, the swab was taken for HSV from vagina and labia and it came back positive for HSV-2. The patient was started on intravenous acyclovir on day 16 of hospitalization. Soon after acyclovir therapy, the platelet count started to improve and after 10 days of antiviral therapy, it improved from 43,000 to 157,000/μL as shown in Figure 1. Also on day 16, cephalexin was stopped and tigecycline and fluconazole were started for empirical coverage of skin fold infection. The patient received a total of 7 days of tigecycline and 10 days of fluconazole therapy. The platelet count mostly remained above 120,000/μL and the patient was later discharged to a nursing home.

## Discussion

A variety of viral, bacterial, and parasitic infections are associated with thrombocytopenia as shown in Table 1 [6]. Different mechanisms have been proposed that contribute to thrombocytopenia in viral infections. Some viruses interact with the receptors on the surface of platelets and interfere with production or increase the destruction. Some viruses infect megakaryocytes and induce apoptosis or interfere with their function by reducing the expression of thrombopoietin receptors [6]. Similarly, a viral infection leads to changes in the structure of the platelet epitope that induces molecular mimicry and triggers immune-mediated destruction. For instance, hepatitis B and C virus infection and human immunodeficiency virus infections cause immune-mediated thrombocytopenia [7]. 

**Table 1:** Common infectious causes of thrombocytopenia [6].

Viral Infections	Bacterial Infections	Parasitic Infections
Chikungunya virus	Brucellosis	Babesiosis
Cytomegalovirus	Disseminated tuberculosis	Leishmaniasis
Dengue	Ehrlichiosis/anaplasmosis	Malaria
Epstein-Barr virus	Helicobacter Pylori	Toxoplasmosis
Hepatitis B virus	Relapsing fever	
Hepatitis C virus	Rickettsia	
Human immunodeficiency virus	Bacteremia/sepsis	
Influenza A virus		
Measles		
Mumps		
Parvovirus B-19 virus		
Varicella-Zoster virus		
Zika Virus		
Rubella virus		

HSV is usually divided into two main types: HSV-1 and HSV-2. Both of which infect epithelial cells of mucocutaneous tissues. HSV-1 typically affects oro-labial tissue whereas HSV-2 typically affects the genital area. However, both strains of the HSV virus can affect oro-labial and genital skin tissues. The clinical manifestation of HSV-2 depends on whether it’s a primary infection, non-primary infection, or recurrent infection and ranges from mild or asymptomatic to severely painful genital ulcers with dysuria, fever, and tender local inguinal lymphadenopathy [8]. Whitaker and Hardison (1978) proposed an immune thrombocytopenia-like state responsible for thrombocytopenia with HSV-2 infection [5]. In our patient, the platelet count dropped gradually as the HSV-2 infection worsened and improved after receiving the treatment with acyclovir. The second drop in platelet count after initial improvement depicts waxing and waning pattern that is frequently associated with immune-thrombocytopenia but lack of response to intravenous steroids suggested that mechanism other than immune-thrombocytopenia is responsible for thrombocytopenia in our patient.

Both vancomycin and piperacillin-tazobactam have shown an association with thrombocytopenia that usually improves with the discontinuation of the therapy [9,10]. In our patient, the discontinuation of both vancomycin and piperacillin-tazobactam failed to improve the thrombocytopenia which makes them unlikely to cause thrombocytopenia. Sepsis is also known to cause thrombocytopenia via different mechanisms such as decreased production, modification of platelet receptors, immune-mediated thrombocytopenia, splenic sequestration, and consumptive coagulopathy [11].

In our patient, the clinical manifestation and microbiology testing were remarkable only for HSV-2 infection. The ulcerations were not associated with painful lymphadenopathy and were unresponsive to cephalosporins which rules out concurrent chancroid ulcers. The clinical condition remained stable and non-septic throughout the hospitalization course, therefore empiric antibiotics or antifungal were less likely to cause the increase of the platelet counts. Normal INR, APTT, fibrinogen level, and the absence of schistocyte on peripheral smear also ruled out sepsis-associated consumptive coagulopathy.

Heparin-induced thrombocytopenia (HIT) is a prothrombotic disorder secondary to antibodies against heparin-platelets factor IV complex. It is an important clinical consideration in hospitalized patients on therapeutic or prophylactic doses of heparin. The onset of platelet count decline within four days of hospitalization, absence of thrombosis or skin necrosis, absence of HIT antibodies, normal serotonin release assay, and lack of improvement in platelet counts with discontinuation of heparin make heparin-induced thrombocytopenia a less likely diagnosis in our patient [12]. Similarly, normal findings on bone marrow biopsy, FISH, and flow cytometry made the diagnosis of hematological malignancy unlikely.

Acyclovir itself has been associated with thrombocytopenia in the literature that usually improves after discontinuation of therapy [13]. However, in our case, the platelet count started to improve soon after the start of treatment with acyclovir which supports our finding that HSV-2 infection was the cause of thrombocytopenia that responded well to antiviral therapy.

Interferon beta-1a, a maintenance agent for relapsing-remitting multiple sclerosis, is associated with transient dose-dependent hematological abnormalities such as neutropenia, leukopenia, and thrombocytopenia. However, thrombocytopenia is typically mild with onset within six months of the start of treatment and resolution within three to four months of continued therapy [14]. Long-term use of interferon beta -1a therapy, with no recent dosage change, combined with the severity of thrombocytopenia makes it a less likely culprit of severe thrombocytopenia. 

The mechanism by which HSV-2 infection brings about thrombocytopenia remains unknown. Further data highlighting the association between HSV-2 infection and thrombocytopenia are needed to enhance our knowledge of the pathophysiological mechanisms of this association.

## Conclusions

HSV-2 infection is not a common cause of thrombocytopenia in hospitalized patients. In the presence of genital ulcers, it is important to consider HSV-2 infection as the cause of thrombocytopenia when other etiologies are already ruled out or improbable. Thrombocytopenia secondary to HSV-2 responds to treatment with acyclovir and treatment should not be delayed.
